# Use of Native Yeast Strains for In-Bottle Fermentation to Face the Uniformity in Sparkling Wine Production

**DOI:** 10.3389/fmicb.2017.01225

**Published:** 2017-06-30

**Authors:** Ileana Vigentini, Shirley Barrera Cardenas, Federica Valdetara, Monica Faccincani, Carlo A. Panont, Claudia Picozzi, Roberto Foschino

**Affiliations:** ^1^Department of Food, Environmental and Nutritional Sciences, Università degli Studi di MilanoMilan, Italy; ^2^Consorzio per la Tutela del FranciacortaErbusco, Italy; ^3^Consorzio Tutela Vini Oltrepò PaveseTorrazza Coste, Italy

**Keywords:** Franciacorta, in-bottle fermentation, Oltrepò Pavese, *Saccharomyces cerevisiae*, sparkling wine, yeast strain selection

## Abstract

The in-bottle fermentation of sparkling wines is currently triggered by few commercialized *Saccharomyces cerevisiae* strains. This lack of diversity in *tirage* yeast cultures leads to a prevalent uniformity in sensory profiles of the end products. The aim of this study has been to exploit the natural multiplicity of yeast populations in order to introduce variability in sparkling wines throughout the re-fermentation step. A collection of 133 *S. cerevisiae* strains were screened on the basis of technological criteria (fermenting power and vigor, SO_2_ tolerance, alcohol tolerance, flocculence) and qualitative features (acetic acid, glycerol and H_2_S productions). These activities allowed the selection of yeasts capable of dominating the in-bottle fermentation in actual cellar conditions: in particular, the performances of FX and FY strains (isolated in Franciacorta area), and OX and OY strains (isolated in Oltrepò Pavese area), were compared to those of habitually used starter cultures (IOC18-2007, EC1118, Lalvin DV10), by involving nine wineries belonging to the two Consortia of Appellation of Origin. The microbiological analyses of samples have revealed that the indigenous strains showed an increased latency period and a higher cultivability along the aging time than the commercial starter cultures do. Results of chemical analyses and sensory evaluation of the samples after 18 months *sur lies* have shown that significant differences (*p* < 0.05) were present among the strains for alcoholic strength, carbon dioxide overpressure and pleasantness, whereas they were not observed for residual sugars content, titratable acidity or volatile acidity. Indigenous *S. cerevisiae* exhibited comparable values respect to the commercial starter cultures. The ANOVA has also proven that the base wine formulation is a key factor, by significantly affecting (*p* < 0.01) some oenological parameters of wine, like alcoholic strength, volatile acidity, carbon dioxide overpressure, titratable acidity and dry extract. The use of native yeast strains for the re-fermentation step can be considered a convenient way for introducing differentiation to the final product without modifying the traditional technology. In a perspective of “precision enology,” where the wine is designed on specific vine cultivars and microorganisms, this work underlines that exploring yeast biodiversity is a strategic activity to improve the production.

## Introduction

The widespread use of selected cultures, commonly found on the market as Active Dry Yeast, is probably the most important innovation that allowed a more effective management of the fermentative process in winemaking since the last century (Pretorius, 2000; Fleet, 2008; Suárez-Lepe and Morata, 2012). Nevertheless, this oenological practice has determined a decrease of diversity in microbial populations involved in fermentation with a consequent reduction of their impact on the sensory characteristics of the final product (Csoma et al., 2010; Di Maio et al., 2012). Actually, it has been widely recognized that each yeast species can contribute to the formation of aromatic compounds through peculiar metabolic pathways and differences in flavor production can be observed at the strain level (Romano et al., 2003; Molina et al., 2009). Despite the high number of starter cultures sold on the market, the available yeast strains are less than what we can think; indeed, manufacturers of different brands often designate the same strain with different codes or names (Fernández-Espinar et al., 2001; Vigentini et al., 2009). The question becomes trickier for the sparkling wines made by the so-called traditional method (*méthode Champenoise*) that require a second in-bottle fermentation of a base wine followed by a prolonged aging over lees. In this case, the commercialized yeast strains are a small number and mostly ascribing to one species, *Saccharomyces cerevisiae* (Torresi et al., 2011; Vigentini et al., 2015; Perpetuini et al., 2016). This condition has led to a widespread homology in organoleptic features of sparkling wines and to a loss of diversity, without exploiting the potential of microorganisms to obtain innovative products by low-aromatic vine cultivars. To overcome these issues, some winemakers used to manage a small amount of must by spontaneous fermentation to enrich the flavor profiles of base wines with the contribution of native yeasts (Vigentini et al., 2014), though the influence of the environmental microorganisms is hardly recognizable. In recent years, many researches have been focused on the selection of indigenous strains to be used as a starter in particular style of wine or in specific regions, with the aim of providing sensory characteristics attributable to the territory of belonging (Capece et al., 2010; Settanni et al., 2012; Suzzi et al., 2012; Tristezza et al., 2012; Rodríguez-Palero et al., 2013; Furdikova et al., 2014; Ilieva et al., 2017). This goal is not easy to carry on for sparkling wine production by traditional method because of the following reasons: first, the starting material is often a mixture of wines and additives (*liqueur de tirage*), formulated by an oenologist according to the cellar style (Pozo-Bayón et al., 2009; Torresi et al., 2011). Besides, the final addition of *liqueur de dosage* can strongly affect the sensory traits (Kemp et al., 2014). Second, several winemakers are convinced that the yeast role in the *prise de mousse* step is only useful for generating the over pressure into the bottle, without significantly influencing the aromatic features. Third, the strain selection for the second fermentation requires long times of testing to verify the effect on characteristics of the sparkling wines and the interactions among environmental and technological factors are difficult to be elucidated (Borrull et al., 2015, 2016).

The in-bottle aging is a complex phenomenon that involves the pivotal roles of the temperature, the base wine formulation and the yeast strain; definitely, an effect on the synthesis and release of aromatic compounds, the cell autolysis, the foaming quality and the bubbling properties of the final product have been demonstrated (Alexandre and Guilloux-Benatier, 2006; Pozo-Bayón et al., 2009; Torresi et al., 2011; Kemp et al., 2014; Perpetuini et al., 2016). In addition, the cellular aptitude to flock is a key point for the selection of strains to be used in traditional method, as it is useful to facilitate the separation process of yeast lees into the bottle by natural settling. The study of genes coding for the flocculent phenotype and their expression in *S. cerevisiae* have revealed the strain specific nature of this property (Tofalo et al., 2014, 2016), even if a high variability in behavior patterns has been observed depending on the environmental conditions and aging time.

The aims of this study were to select indigenous yeast strains throughout consecutive screening steps based on technological and qualitative criteria for sparkling wine-making and to compare the fermentative performances of these strains with those already used by the wine industry in real cellar situations. In particular, we performed the experimental trials at nine wineries of Franciacorta and Oltrepò Pavese areas in Lombardy region, which is the largest Italian district where sparkling wines are produced by traditional method (Vigentini et al., 2014; Foschino et al., 2015), through the involvement of the respective consortia of Appellation of Origin.

## Materials and methods

### Strain collection

One hundred and thirty three *S. cerevisiae* strains identified and genotyped in a previous work (Vigentini et al., 2015), were chosen based on their distinctive inter-delta profiles obtained by capillary electrophoresis. Fresh cultures of each strain grown in YPD broth (10 g/L yeast extract, 20 g/L peptone, 20 g/L glucose, pH 6.5) at 26°C, for 24 h in orbital agitation (120 rpm), were centrifuged at 3,500 *g* for 15 min and then the cells were resuspended in the same broth added with 25% (v/v) glycerol. Cell suspensions were stored at −80°C or on YPD agar (18 g/L) at 4°C for short-term storage.

### Selection for oenological traits

Technological characteristics like fermenting power, fermenting vigor and resistance to sulfur dioxide, were preliminarily investigated in order to select strains with oenological potential for sparkling white wine production according to the OIV-OENO Resolution, 370-2012 (2012).

The fermenting power, expressed as % (v/v) ethanol produced, was daily evaluated by monitoring the weight loss for 3 weeks at 18°C in YPD broth added with 260 g/L glucose in static conditions. A 250 mL flask, sealed with a Müller trap and containing 100 mL of the growth broth, was inoculated with 1% (v/v) of a fresh culture, realized as previously described, in order to obtain approximately 1 × 10^6^ CFU/mL starting concentration. The fermenting vigor, expressing the speed at which yeast starts the fermentation, was determined as grams of CO_2_ lost in 48 h from the beginning of the trial. The resistance to sulfur dioxide was examined by spotting 5 μL of fresh culture, onto YPD plates acidified at pH 3.5 with tartaric acid and added with 15 g/L agar. Variable amounts of sterile solution of potassium metabisulfate were previously supplemented to the medium in order to obtain doses of total SO_2_ ranging from 100 to 300 mg/L. Resistance degree to sulfur dioxide was reported as the maximum dose at which the yeast exhibited an evident growth after incubation at 26°C for 72 h. A control test without adding solution of potassium metabisulfate was carried out.

A second step of investigation, limited to the strains that passed the first screening phase, was carried out on the characteristics that influence the wine quality like acetic acid, glycerol and hydrogen sulfide productions. The acetic acid and glycerol amounts were assayed in the supernatants at the end of the fermentations of the first set of analysis. Two mL aliquots of cell cultures were centrifuged at 3500 *g* for 15 min and specific enzymatic kits based on spectrophotometric UV method were used (Jenway, UV-visible spectrophotometer, model 7315, Bibby Scientific Limited, Stone, UK), according to the supplier's recommendations (Megazyme International, Bray, Ireland). The synthesis of hydrogen sulfide was estimated by spotting 5 μL of a fresh culture, on BIGGY agar plates (Oxoid limited, Basingstoke, UK). After incubation at 26°C for 72 h the color of the colonies may range from white-cream until brown-black in function of increasing amounts of hydrogen sulfide produced.

A third step of selection, limited to those strains that passed the second screening phase, was performed by assessing the ability of cells to grow in presence of ethanol and by characterizing the flocculent phenotype. The alcohol tolerance test was performed in 100 mL bottles with 75 mL YEPD broth acidified at pH 3.5 with tartaric acid and containing 10% ethanol (v/v), by inoculating a 1% (v/v) of a fresh culture in order to realize an approximately 1 × 10^6^ CFU/mL starting concentration. After inoculation, samples were incubated at 15°C in static conditions and cell growth was monitored every 5 days by Optical Density measurements at 600 nm in U.V-Visible spectrophotometer (Jenway). Flocculation test was carried out according to the protocol of Suzzi and Romano (1991) with some modifications: after the evidence of cell growth (OD_600 nm_ > 1.0) in samples used for the alcohol tolerance test, 3 mL of microbial suspension were taken from there, centrifuged at 2,000 *g* per 5 min and the pellet was resuspended in 3 mL of flocculation buffer (50 mmol/L Na acetate/acetic acid, 5 mmol/L CaSO_4_, pH 4.5). The OD_600 nm_ values were immediately measured and after 15 min, by leaving the cuvette at room temperature in a static position. The degree of flocculence for each strain was calculated as follows: F = OD _600 nm after 15 min_ / OD _600 nm at starting time_ per 100 with scores ranging 0–10 (very flocculent, corresponding to point 4 of Suzzi and Romano's scale), 10–30 (moderately flocculent, point 3), 30–70 (weakly flocculent, point 2), 70–90 (poorly flocculent, point 1), 90–100 (non-flocculent, point 0).

### Set up of the *Tirage* experiments

Based on the results previously obtained, four strains (FX and FY isolated in Franciacorta, OX and OY isolated in Oltrepò Pavese areas) were selected to be used as starter cultures for the re-fermentation trials of base wines in nine different wineries (Table 1). Each tested strain was pre-inoculated in 20 mL of YEPD broth at 26°C for 24 h in shaking state at 120 rpm; then 2 mL of this culture were transferred to 500 mL polycarbonate Erlenmeyer flasks with DuoCAP® (TriForest, Irvine, USA), containing 200 mL of YEPD broth, and incubated at 26°C for 48 h in orbital agitation (120 rpm). After OD measurement at 600 nm, a volume corresponding to a concentration of 5 × 10^9^ cells per mL was centrifuged (Hettich Zentrifugen, Rotina 380 R, Germany) at 3,500 *g* for 15 min; the pellets were then resuspended in 25 mL of YEPD broth and stored at 4°C. The same protocol was carried out for the strains of the commercial starter culture habitually utilized in the relative cellar (Table 1), in order to compare the performances under the same conditions. Each cellar used its own base wine, prevalently made with Chardonnay cultivar for Franciacorta wineries and Pinot Noir for Oltrepò Pavese ones (Table 1). In each winery three trials were performed in parallel, two by inoculating the indigenous strains of the corresponding territory and one with the usual starter culture strain (Table 1). For each trial, 50 L of clarified base wine, with different ethanol content (Table 1), was added with approximately 24 g/L of sucrose and sterilized by filtering. A *pied de cuvee* for each tested strain was prepared by the following steps: 25 mL of the previously concentrated cell suspension were diluted in 250 mL of sterile distilled water, pre-warmed at 30°C; after 30 min, 250 mL of base wine, pre-warmed at 24°C, were added and maintained at the same temperature in a thermostatic room; after 4 h, 500 mL of base wine, pre-warmed at 24°C, and 1 g of yeast autolysate containing ammonium salts, amino acids, thiamine and pantothenic acid (Proteofast, BioEnologia 2.0 S.r.l., Oderzo, Treviso, Italy) were added and thoroughly mixed; after 4 h, 500 mL of base wine, pre-warmed at 24°C, and 2 g of yeast autolysate were added and thoroughly mixed. After a night at 24°C, 1 L of base wine at 20°C and 4 g of yeast autolysate were added and thoroughly mixed. Lastly, the whole *pied de cuvee* (2.525 L) was poured into the 50 L base wine mass, added with 30 mL of adjuvant 83 Liquide (Station Oenotechnique de Champagne, Magenta, France) and mixed thoroughly to form the *liqueur de tirage*. For each trial, approximately 70 Champagne bottles (750 mL type) were filled and equipped with plastic caps (*bidules*), sealed with crown caps and maintained at cellar temperature for 18 months in each cellar.

**Table 1:** Information about wine-making of experimental sparkling wine samples.

**Winery**	**Vine-growing area**	**Prevalent grape cultivar in base wine**	**Ethanol content (% v/v) in base wine**	**Indigenous selected strains**		**Starter culture strain habitually inoculated**
I	Franciacorta	Chardonnay	10.5	FX	FY	IOC18-2007^†^
II	Franciacorta	Chardonnay	11.5	FX	FY	Not disclosed
III	Franciacorta	Chardonnay	11.5	FX	FY	IOC18-2007
IV	Franciacorta	Chardonnay	11.0	FX	FY	EC1118^‡^
V	Franciacorta	Chardonnay	11.0	FX	FY	DV10^‡^
VI	Oltrepò Pavese	Pinot noir	11.0	OX	OY	IOC18-2007
VII	Oltrepò Pavese	Pinot noir	11.0	OX	OY	IOC18-2007
VIII	Oltrepò Pavese	Pinot noir	11.0	OX	OY	EC1118
IX	Oltrepò Pavese	Croà	10.5	OX	OY	DV10

† *Institut Oenologique de Champagne, Épernay, France*.

‡ *Lalvin®, Lallemand Oenology, Petaluma, USA*.

### Monitoring the *prise de mousse* experiments by microbiological analysis

The trend of the second fermentation was monitored for each trial by sampling two bottles at the starting time, after 2 weeks and then every month until the fourth one. Cell concentration was determined for each sample by plate count technique (OIV-OENO Resolution, 206/2010, 2010). After appropriate dilution in Peptoned Water (Merck, Germany) 100 μL of sample were spread onto WL agar plates (Merck) and incubated at 25°C for 3 days. Then, after counting, up to four colonies grown in plates at the highest dilutions were randomly isolated by twice streaking, in order to identify the dominant strains through a molecular typing technique. DNA extraction was carried out according to the protocol of Vigentini et al. (2014) and the amplification of inter-delta regions (δ-PCR) was performed to discriminate the isolates (Legras and Karst, 2003). After electrophoretical separation as reported by Vigentini et al. (2014), the obtained inter-delta profiles were analyzed using Quantity One Software (Bio-Rad, CA, USA).

Cell viability was estimated by microscopic technique after applying a methylene blue staining (OIV-OENO Resolution, 206/2010, 2010). Appropriate dilutions of the samples were observed in a Burker counting chamber at a magnification of 400 X (Microscope Standard 25, Zeiss, Germany), within 15 min contact with the stain. Cell viability was expressed as the percentage ratio between the number of the not stained cells (live) and the number of the total observed cells.

### Chemical analysis

At the end of the aging *sur lies* (18 months), two bottles of wine samples for each trials were analyzed for: alcoholic strength (% v/v), glucose and fructose content (g/L), titratable acidity, expressed as tartaric acid (g/L), volatile acidity, expressed as acetic acid (g/L) and total sulfur dioxide (mg/L) according to the standard protocol proposed by OIV (2014). Carbon dioxide overpressure (bar) was measured in one sample per cellar by aphrometric technique (OIV, 2014).

### Sensory evaluation

The sensory evaluation was performed in different sessions on bottled samples at 18 months of aging *sur lies* by a panel of at least 10 skilled judges working at the wineries involved in the project or collaborating with the wine consortia. Yeast precipitates (lees) were previously removed from the tested samples by riddling and disgorging operations; *liqueur d'expedition* was not added. The wine quality was estimated by defining aroma descriptors that were chosen by the taster panels in a previous session according to the rules of respective Appellation of Origin Committees, Consorzio Franciacorta (http://www.franciacorta.net/en/) and Consorzio Tutela Vini Oltrepò Pavese (http://www.vinoltrepo.org/it/eng/). Samples were presented in a blind randomized sequence. Then judges were asked to score the samples on a scale of a pleasantness distributed on a decimal scoring, where point 0 meant extremely unpleasant and point 10 extremely pleasant, by considering the odorous characteristics and the taste, separately.

### Statistical analysis

The effect of some factors, such as the yeast strain inoculated for the developing of the second fermentation, the wine base formulation and cellar conditions, the prevalent grape cultivar worked for the base wine preparation, on some chemical parameters and sensorial evaluations were investigated by one-way ANOVA (Camussi et al., 1986) according to the general linear model. Results of microbiological counts were transformed in the respective decimal logarithms to match a normal distribution of values. Data were processed with Statgraphic® Plus 5.1 for Windows (StatPoint, Inc., Herndon, Virginia, USA). When the effect was significant (*p* < 0.05), differences between means were separated by LSD test of multiple comparisons.

## Results

### Strain selection

**Figure 1:**
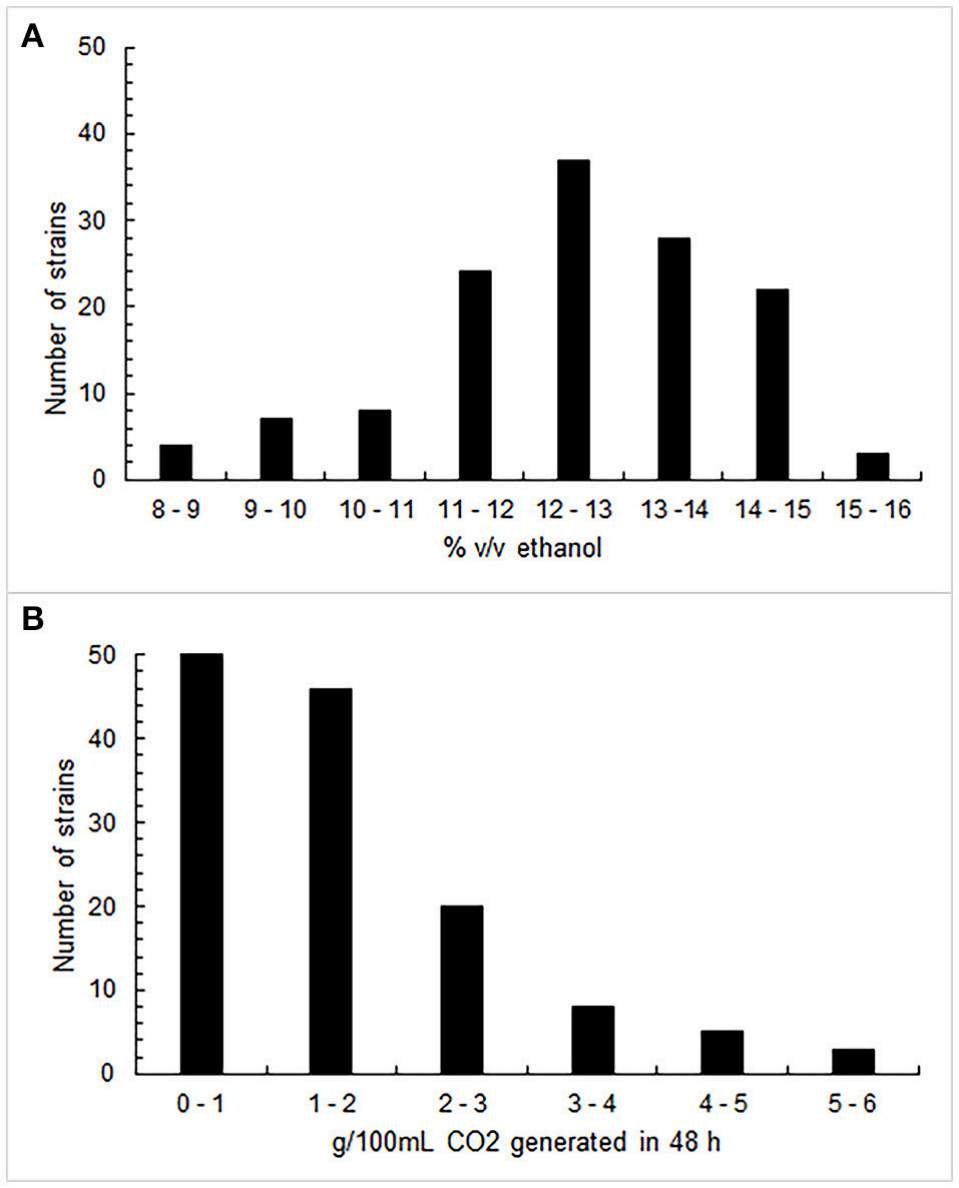
Distribution of fermenting power values **(A)**, expressed as % (v/v) ethanol, and of fermenting vigor values **(B)**, expressed as CO_2_ g/100 mL generated in 48 h, by 133 *S. cerevisiae* strains.

Figure 1 shows the results obtained through the fermenting power (A) and the fermenting vigor (B) assays. Two-thirds of the 133 investigated strains proved to be able to produce more than 12% (v/v) alcohol in the tested conditions, with an arithmetic mean of 12.4% (v/v). In particular, 68 strains exceeded the median value of 12.6% (v/v). Generally, the *S. cerevisiae* isolates did not exhibit a high fermenting vigor since the average value was 1.53 g of CO_2_ per 100 mL within 48 h, even if 27% of them generated more than 2 g; 65 strains were those that overcame the median value of 1.15 g. As regards the tolerance test to sulfur dioxide, 97, 78, and 29% of strains could grow at 100, 200, and 300 mg/L of total SO_2_,respectively. Consequently, 46 strains that simultaneously displayed to exceed the median value of the fermenting power, the median value of the fermenting vigor and the high value of resistance to sulfur dioxide, were selected for the next phase of screening.

**Figure 2:**
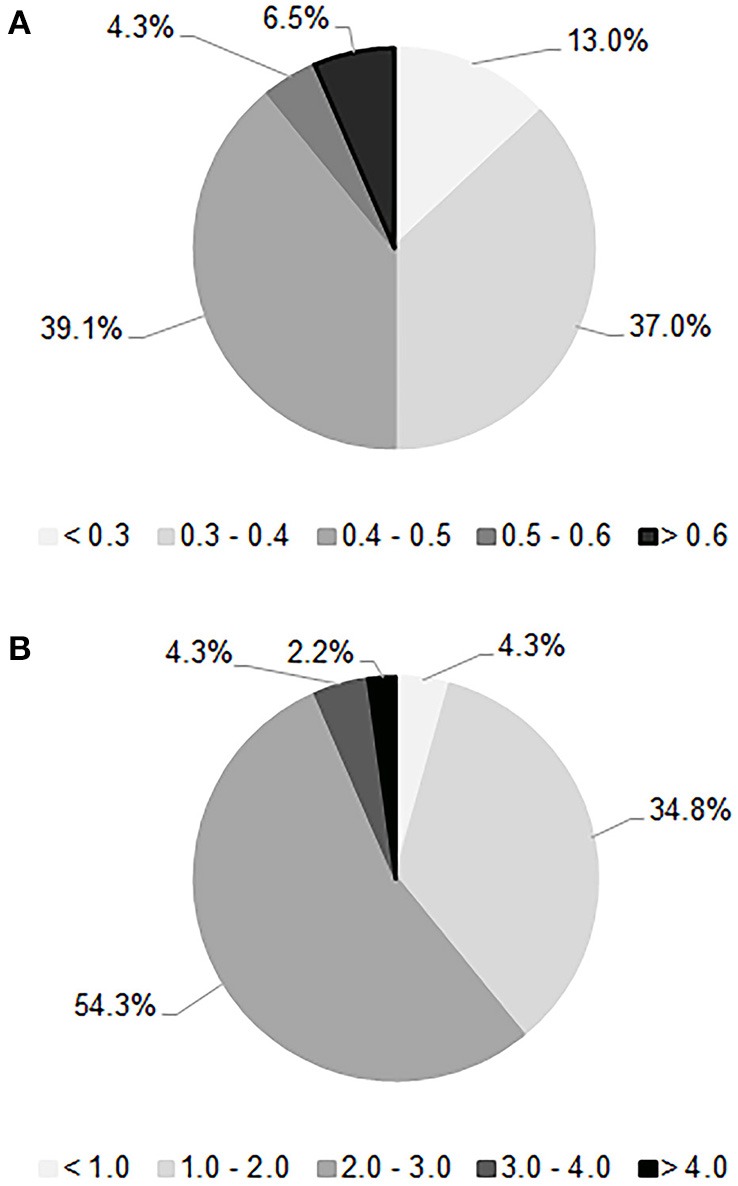
Distribution of the acetic acid **(A)** and glycerol **(B)** amounts, expressed as g/L, produced by 46 *S. cerevisiae* selected strains after the first phase of screening.

Quantification assays of the acetic acid and glycerol productions are shown in Figure 2. The mean value of the acetic acid production was 0.41 g/L. In the tested conditions, only six strains developed a low level of volatile acidity (<0.3 g/L), which is crucial for sparkling wine quality. On the other hand, the average amount of glycerol production was 2.18 g/L, a low value compared to the data reported in literature (Scanes et al., 1998; Suárez-Lepe and Morata, 2012); only three strains proved to be able to generate more than 3 g/L of glycerol. The hydrogen sulfide test revealed that 78% of strains were high synthesizers of this compound since they generated brown colonies, while 20% were low producers with formation of beige-cream colored colonies; only one strain did not produce hydrogen sulfide. The choice was oriented toward the lowest producers of acetic acid and H_2_S and highest producers of glycerol. Based on these outcomes, 16 strains were taken for the next step of selection consisting of alcohol tolerance and flocculation tests. All of them reached an OD_600nm_ > 1.0 within 10 days of incubation at 15°C in the acidified medium added with ethanol at 10% (v/v), by demonstrating reliability to start the second fermentation. As regards the flocculation test only one strain showed a degree of flocculence of point 2, while the others proved to be poorly flocculent (point 1) in 13% of cases or non-flocculent phenotype (point 0) in 81%. Thus, two strains isolated in both investigated territories, named FX and FY from Franciacorta area and OX and OY from Oltrepò Pavese area that presented the best scores in the all considered parameters were designated for the in-bottle fermentation trials.

### Monitoring of the in-bottle fermentation trials

**Figure 3:**
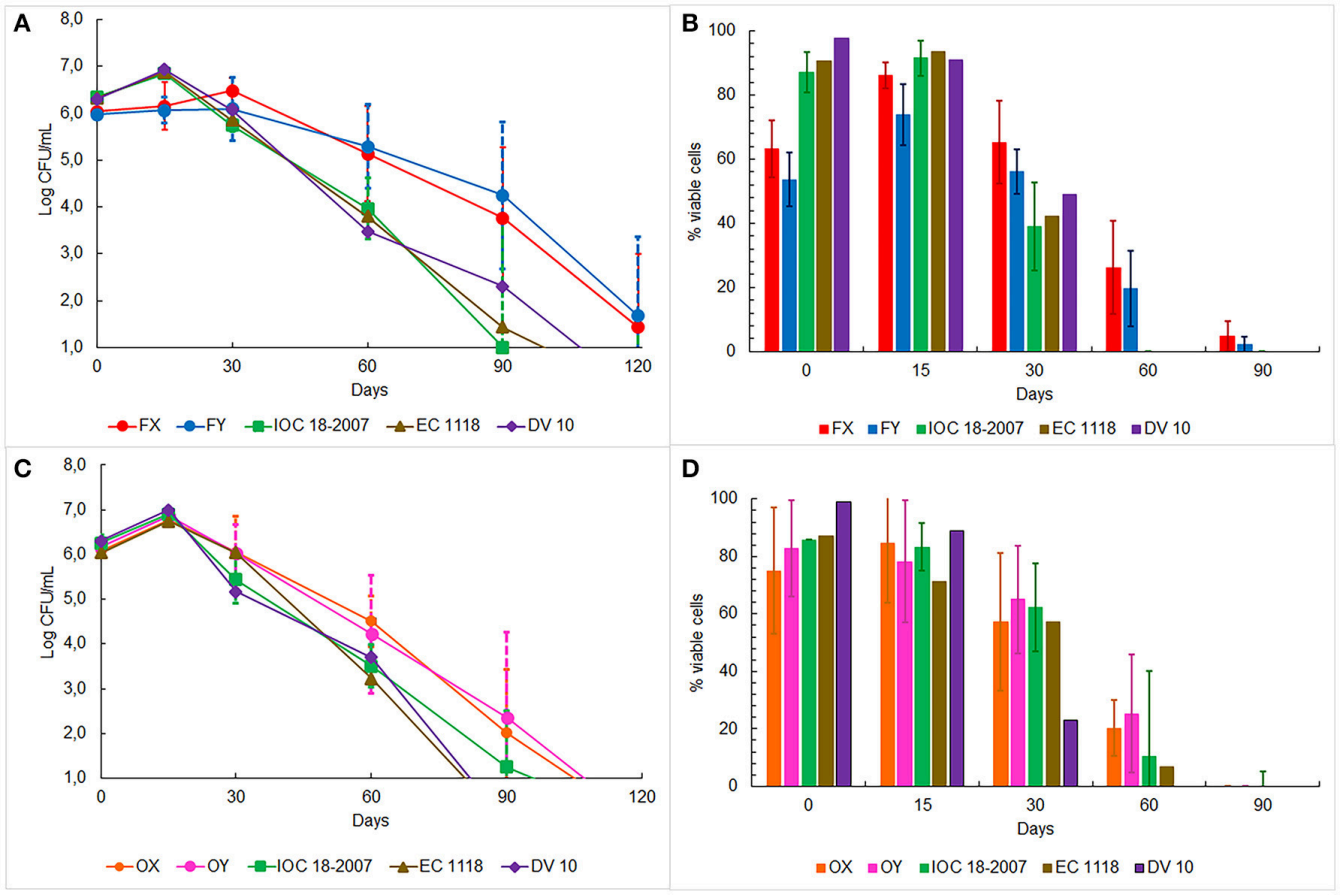
Monitoring of yeast plate counts in base wine samples inoculated with: **(A)** FX and FY strains from Franciacorta area, **(C)** OX and OY strains from Oltrepò Pavese area and other commercial strains. Yeast cell viability in the same base wine samples inoculated with: **(B)** FX and FY strains from Franciacorta area **(D)** OX and OY strains from Oltrepò Pavese area and commercial strains.

The oenological performances of the four selected strains were tested in *prise de mousse* experiments after *tirage* operation carried out in 750 mL bottles. Five Franciacorta wineries, for the FX and FY strains, and four Oltrepò Pavese wineries, for the OX and OY strains, were involved in the experimental plan according to the decision of the winemakers Consortia (Table 1). The starter culture IOC18-2007, EC1118 or Lalvin DV10, which was habitually used by the single cellar, was prepared in the same conditions as the indigenous strain and it was chosen as control test (Table 1). The average temperature of the cellars was 14.5°C ± 2°C. Samples were analyzed by determining cell counts, cell vitality and genetic identification of the dominant strains. The cell concentration in the samples inoculated with FX and FY strains showed similar trends (Figure 3A), by unveiling a slower increase in plate counts at the beginning of the trials respect to the control tests inoculated with the commercial starter cultures. Furthermore, the enumeration of cultivable cells of both Franciacorta indigenous strains remained higher than 5 Log CFU/mL at 2 months and approximately at 4 Log CFU/mL after 3 months of aging, exhibiting significant different log counts (*p* < 0.05) respect to the references strains. After 4 months IOC18-2007, EC1118 and Lalvin DV10 strains were no longer detectable by plate count technique (Figure 3A). Microscopic observations revealed that lower ratios of viable cells were present in the bottles inoculated with FX and FY strains in comparison to those prepared with the conventional yeasts, up to 2 weeks of incubation. Conversely, about 20% of cell population of indigenous Franciacorta strains remained metabolically active until 2 months, which was not the case for the common starter cultures (Figure 3B). After 120 days, 100% of cells appeared not viable for any strains. The analysis of the DNA amplification profiles of the inter-delta regions confirmed the dominance of the inoculated strains for each trial and all along the aging period, until it was possible to isolate colonies (data not shown).

The cell concentration in the bottles elaborated in Oltrepò Pavese cellars displayed a homogeneous behavior since no significant difference came out from the samples inoculated with the different yeasts. However, OX and OY strains always preserved a cultivability higher than the commercial starter cultures of approximately one order of magnitude from 1 to 4 month (Figure 3C). The results of staining test evinced comparable percent values of cell viability for both Oltrepò Pavese indigenous strains and the conventional ones throughout the monitored period. For all samples, after 120 days it was no longer possible to find colonies, whereas after 90 days viable cells could not be observed anymore (Figure 3D). The genotypic patterns obtained from δ-PCR analysis allowed to establish that the inoculated strains persisted as dominant yeast population in each trial during the aging time (data not shown).

### Quality evaluation of sparkling wine samples

The mean values and relative standard deviation of some oenological parameters obtained from the chemical and sensory analyses of the experimental samples for different strains and different wineries are reported in Tables 2, 3, respectively. Data were subjected to one-way ANOVA in order to evaluate the effect of the “strain” inoculated for the second in-bottle fermentation, the “winery” factor, intended as the set of additives and cellar environment related to wine-making operation, or the “grape” variety, mainly used to produce the base wine. As regards the “strain” factor (Table 2), the average datum of alcoholic strength in sparkling wines inoculated with OX strain was significantly lower (*p* < 0.05) than those inoculated with FX, OY, EC1118 and DV10 strains. Similarly, the pressure of carbon dioxide reached inside the bottles inoculated with FX, FY and DV10 strains was significantly higher (*p* < 0.05) than that inoculated with OX and OY. Conversely, no significant differences were observed among mean values in residual sugars content (g/L), or titratable acidity (g/L), or volatile acidity (g/L). Also the average data of total SO_2_ (mg/L) and dry extract (g/L) did not reveal significant differences among the samples inoculated with different strains. The results of sensory test for the smell pleasantness gave significantly (*p* < 0.05) higher scores to the samples re-fermented with strains FX, FY, OY, and DV10. The values obtained from the tasting evaluation confirmed a higher agreeableness (*p* < 0.05) for the sparkling wines inoculated with strains FX, FY and DV10. The “winery” factor (Table 3) proved to be heavily engaged by determining significant differences in alcoholic strength (*p* < 0.01), residual sugars content (*p* < 0.05), volatile acidity (*p* < 0.01) and CO_2_ overpressure (*p* < 0.01). Also the average data of titratable acidity (*p* < 0.01), total SO_2_ (*p* < 0.05) and dry extract (*p* < 0.01) revealed important differences among samples prepared in different wineries, showing that the formulation of the base wine, the cellar practices and the environmental conditions deeply affected the outcomes. The results of the sensory evaluation confirmed the substantial impact of how the wine-making was carried out in the single cellar; indeed significant differences were found among the scores that were attributed to the samples of each winery, by displaying *p* < 0.01 for the perception of volatile compounds and *p* < 0.05 in the case of taste sensations. Finally, the factor “grape cultivar” used to prepare the base wine seemed to significantly influence the following parameters: residual sugars amount (*p* < 0.01), where the samples prevalently made with *Chardonnay* and *Pinot Noir* showed mean values of 1.03 and 1.36 g/L respectively, vs. a mean value of 2.87 g/L for those prepared with *Croà*; level of total SO_2_ (*p* < 0.05) with averages data of 44, 35 and 51 mg/L for *Chardonnay, Pinot Noir* and *Croà* wines, respectively; carbon dioxide overpressure (*p* < 0.01) since the mean value in *Chardonnay* based samples (7.1 bar) was higher than in those of *Pinot noir* (5.7 bar) and *Croà* (5.8 bar). Both the scores obtained in sensory tests for olfactive and gustative pleasantness from sparkling wines prevalently produced with *Chardonnay* variety resulted higher (*p* < 0.05) than those made with *Pinot Noir* and *Croà*.

**Table 2:** Mean (± standard deviation) of oenological parameters obtained from sparkling wine samples, inoculated with different strains, after 18 months of aging on the lees in nine cellars of Franciacorta and Oltrepò Pavese areas.

**Strain**	**FX**	**FY**	**OX**	**OY**	**IOC 18-2007**	**EC 1118**	**DV10**
Winery	I, II, III, IV, V	I, II, III, IV, V	VI, VII, VIII, IX	VI, VII, VIII, IX	I, III, VI, VII	IV, VIII	V, IX
Alcoholic strength (% v/v)	12.5^b^ (± 0.5)	12.3^a,b^ (± 0.6)	11.9^a^ (± 0.4)	12.5^b^ (± 0.3)	12.3^a,b^ (± 0.6)	12.5^b^ (± 0.1)	12.7^b^ (± 0.2)
Glucose and fructose content (g/L)	0.9 (± 0.7)	1.4 (± 1.0)	2.2 (± 1.9)	1.9 (± 1.1)	0.5 (± 0.2)	1.5 (± 0.3)	0.7 (± 0.5)
Titratable acidity (g/L)	7.2 (± 0.6)	6.9 (± 0.6)	6.7 (± 0.8)	6.7 (± 1.1)	7.2 (± 0.5)	6.1 (± 0.4)	6.9 (± 0.7)
Volatile acidity (g/L)	0.41 (± 0.15)	0.45 (± 0.20)	0.56 (± 0.12)	0.43 (± 0.05)	0.42 (± 0.09)	0.58 (± 0.18)	0.41 (± 0.07)
Total SO_2_ (mg/L)	44 (± 19)	44 (± 20)	40 (± 17)	39 (± 10)	32 (± 5)	46 (± 15)	63 (± 15)
Dry extract (g/L)	18.6 (± 1.1)	18.7 (± 1.1)	19.3 (± 1.7)	18.8 (± 1.1)	18.6 (± 1.4)	18.6 (± 0.4)	19.6 (± 0.6)
CO_2_ overpressure (bar)	7.2^d^ (± 0.5)	6.8^c,d^ (± 0.8)	5.6^a^ (± 0.5)	5.9^a,b^ (± 0.5)	6.4^a,b,c^ (± 1.1)	6.3^a,b,c^ (± 0.9)	6.8^b,c,d^ (± 1.0)
Olfactive pleasantness	6.0^a^ (±1.3)	5.8^a^ (±1.2)	5.0^b^ (±1.1)	5.7^a^ (±1.6)	5.4^a,b^ (±1.8)	5.6^a,b^ (±1.2)	5.9^a^ (±0.9)
Gustative pleasantness	5.9^a^ (±1.7)	5.8^a^ (±1.7)	5.4^a,b^ (±1.4)	4.9^b^ (±1.6)	5.4^a,b^ (±1.7)	4.6^b^ (±1.7)	6.0^a^ (±1.2)

*Values on the same row with different superscripts letters are significantly different (P ≤ 0.05)*.

**Table 3:** Mean (± standard deviation) of oenological parameters obtained from sparkling wine samples, made in different wineries of Franciacorta and Oltrepò Pavese areas, after 18 months of aging on the lees of different strains.

**Winery**	**I**	**II**	**III**	**IV**	**V**	**VI**	**VII**	**VIII**	**IX**
Strains	FX, FY, IOC 18-2007	FX, FY, strain not disclosed	FX, FY, IOC 18-2007	FX, FY, EC1118	FX, FY, DV10	OX, OY, IOC 18-2007	OX, OY, IOC 18-2007	OX, OY, EC1118	OX, OY, DV10
Alcoholic strength (% v/v)	11.7^a^ (± 0.3)	13.0^e^ (± 0.2)	12.8^d,e^ (± 0.2)	12.3^b,c^ (± 0.3)	12.4^b,c^ (± 0.4)	12.5^c,d^ (± 0.4)	12.2^b,c^ (± 0.2)	12.4^b,c^ (± 0.3)	12.0^a,b^ (± 0.6)
Glucose and fructose content (g/L)	1.5^a,b^ (± 1.0)	0.4^a^ (± 0.3)	0.8^a,b^ (± 0.5)	1.8^b,c^ (± 0.5)	0.7^a,b^ (± 0.5)	0.6^a^ (± 0.6)	0.7^a,b^ (± 0.7)	2.7^c^ (± 1.3)	2.8^c^ (± 1.8)
Titratable acidity (g/L)	6.7^b^ (± 0.4)	7.0^c^ (± 0.3)	7.5^d^ (± 0.2)	6.5^b^ (± 0.2)	7.8^e^ (± 0.3)	7.9^e^ (± 0.6)	7.2^c^ (± 0.4)	5.9^a^ (± 0.4)	6.6^b^ (± 0.4)
volatile acidity (g/L)	0.48^c^ (± 0.10)	0.36^a,b^ (± 0.07)	0.29^a^ (± 0.07)	0.69^d^ (± 0.09)	0.37^a,b^ (± 0.07)	0.42^b,c^ (± 0.04)	0.51^c^ (± 0.09)	0.50^c^ (± 0.08)	0.50^c^ (± 0.16)
Total SO_2_ (mg/L)	30^a,b^ (± 8)	40^c^ (± 7)	25^a^ (± 6)	56^d^ (± 6)	72^e^ (± 8)	33^a,b,c^ (± 6)	37^b,c^ (± 4)	36^b,c^ (± 5)	51^d^ (± 6)
Dry extract (g/L)	18.6^b,c^ (± 0.6)	17.8^a,b^ (± 0.5)	19.8^d^ (± 0.6)	18.2^b,c^ (± 0.5)	19.7^d^ (± 0.6)	19.8^d^ (± 1.1)	17.3^a^ (± 0.6)	18.8^c^ (± 0.5)	19.8^d^ (± 0.8)
CO_2_ overpressure (bar)	7.0^d^ (± 0.4)	7.7^e^ (± 0.3)	7.4^d,e^ (± 0.4)	6.3^c^ (± 0.8)	7.2^d,e^ (± 0.5)	6.3^c^ (± 0.4)	5.8^b,c^ (± 0.4)	5.6^a,b^ (± 0.4)	5.2^a^ (± 0.4)
Olfactive pleasantness	5.9^b,c^ (±1.0)	5.9^b,c^ (±1.3)	6.2^c^ (±1.2)	5.9^b,c^ (±1.2)	5.9^b,c^ (±1.3)	6.1^c^ (±1.4)	4.4^a^ (±1.5)	5.2^b^ (±0.9)	5.4^b,c^ (±0.9)
Gustative pleasantness	5.9^c,d^ (±1.6)	4.8^a^ (±1.5)	5.8^b,c,d^ (±1.5)	5.9^c,d^ (±2.2)	6.4^d^ (±1.6)	5.5^a,b,c^ (±1.8)	5.1^a,b^ (±1.5)	4.8^a^ (±0.9)	5.2^a,b^ (±1.2)

*Values on the same row with different superscripts letters are significantly different (P ≤ 0.05)*.

## Discussion

The previous results of an investigation (Vigentini et al., 2015) on the indigenous microbiota in wine-making environment of Franciacorta and Oltrepò Pavese areas, have revealed a high level of genomic diversity within the species *S. cerevisiae*, through polymorphism analysis of the interdelta regions by capillary electrophoresis. Likewise, in this work, the determination of some phenotypic characteristics on the same *S. cerevisiae* strains have confirmed the presence of a large range of values in metabolite production, such as fermenting power, fermenting vigor, acetic acid, glycerol, and hydrogen sulfide, or in resistance to sulfur dioxide. The observation of this intraspecific biodiversity provides a wealth for the potential exploitation to obtain strains tailored to the needs of the wine producer (Pretorius, 2000; Fleet, 2008). Anyway, the adoption of selection criteria results in a hard activity when the strains to be investigated are hundreds, since the priorities planning and the choice of the tasks to achieve the goals become conclusive. In the present work, a polyphasic approach was carried out by considering each strain and the overcoming of the threshold of the median value for some oenological parameter per each phase of the study. Primarily, the selection has been addressed to *S. cerevisiae* as it is considered the most capable yeast species to realize a secondary fermentation starting from high alcohol concentration and in the presence of sulfur dioxide. The second selection occurred for the strains that showed values higher than the median ones for other quality parameters important for sparkling wine-making, such as the low production of acetic acid, high production of glycerol, and low formation of hydrogen sulfide. Again, those strains that have exceeded the median values were chosen for the evaluation of the resistance to ethanol and the flocculent phenotype. Finally, in order to meet a request of the Appellation of Origin Committees of the winemakers, the belonging to the territory was the last criterion used to decide which strains should be tested in *prise de mousse* trials under actual cellar conditions.

The experimental plan stated that each winery had to perform the in-bottle fermentation experiments inoculating its own base wine with the two selected indigenous strains, isolated in the relevant vine-growing area, *plus* the starter strain normally used in its own cellar, according to a protocol previously planned and shared with the oenologists. This allowed us to compare the data obtained from different strains in the same operative conditions, as well as to evaluate the outcome from the same strain in different wineries by assessing its performance in different environments under real operative conditions.

As regards the cell counts and the strain identification, the results reveal that all selected strains are capable of developing and dominating the in-bottle fermentation. However, it should be noted that the Franciacorta indigenous strains show an increased latency period and a higher cultivability than the others along the aging time do. The natural autolysis of yeast, which can be estimated by the drop in percent cell viability, occurred after 2/3 months from the inoculation time, as expected at this temperature (Alexandre and Guilloux-Benatier, 2006).

The ANOVA of results from the analyzed samples at the end of the aging time (18th month) evinces that significant differences among the strains are present for some oenological parameters like the final alcohol content, the achieved carbon dioxide pressure and the sensorial traits. Interestingly, the indigenous strains get a valuation comparable to the one of the conventional starter cultures, or superior as in the case of FX strain from Franciacorta area. This confirms that the strain is a key element affecting the quality of the product, also in sparkling wine by traditional method, as already reported by few authors (Martínez-Rodríguez et al., 2002; Martí-Raga et al., 2016). Nevertheless, the comparison of data observed in samples managed in different cellars with the same strain proves that the “winery,” described as the set of the base wine formulation and the environmental conditions, is the most conditioning factor since significant differences are found in all investigated oenological parameters. These data sustain how much the oenologist's choices are fundamental in selecting the ingredients, assembling the *cuvée* and managing the cellar practices for the quality of the final product. For some parameters, also the prevalent grape cultivar used to make the base wine appears to significantly influence the characteristics of the sample wines.

Although some oenological aspects were not considered in this work, this investigation demonstrates the possibility of recovering indigenous *S. cerevisiae* strains in the environment, that exhibit technological and quality traits suitable for the traditional method, especially the pursuing of the in-bottle fermentation at low temperature starting from a high alcohol content.

Increasing the choice of available strains meets the needs of the sparkling wines industries directed toward an expanding global market searching a differentiation of sensory quality and a recognition of a link with the territory of production. Indeed, the change of the yeast for the second fermentation can be easily introduced to improve or to obtain a typicality of the product without modifying the traditional technology (Pozo-Bayón et al., 2009; Kemp et al., 2014). This goes in the direction of an enology of precision where the wine is designed by combining the specific vine cultivar with a peculiar technology and exploiting the potential metabolic activities of specific microorganisms; over all that it is true for non-aromatic varieties (Vigentini et al., 2016), as in many sparkling wine productions.

Finally, the natural occurrence of native alcohol-tolerant yeasts in the environment may leads the oenologist toward the design of innovative procedure for sparkling wine-making, in order to maximize the potential of microbial diversity present in the current vintage or belonging to the territory. As suggestion, it could be possible to make spontaneous fermentation in a volume of selected must from healthy grapes and, at the end of fermentation, to collect the indigenous microbial populations by centrifugation. Then, this part containing the natural mixture of high ethanol resistant strains could be re-inoculated as starter culture into the base wine for the *tirage* operation.

## Author contributions

IV contributed to the design and organization of the work, to the management of lab work, to the data collection and analysis, to draft and review the manuscript; SB and FV contributed to the microbiological, chemical, molecular and sensory analyses of the samples, to the assistance to cellar work at the wineries and to draft the manuscript; MF and CAP, each for its own Consortium, contributed to the design of the work, to the management of cellar work at the wineries and to the organization of sensory sessions; CP contributed to the management of lab work, to the data collection and analysis, to draft the manuscript; RF contributed to the design and organization of the work, to the data collection and analysis, to draft and review the manuscript, and ensured that all questions related to the accuracy or integrity of any part of the work were appropriately investigated and resolved.

### Conflict of interest statement

The authors declare that the research was conducted in the absence of any commercial or financial relationships that could be construed as a potential conflict of interest.
